# Health complaints before and at one and five years after removal of dental amalgam restorations – data from a prospective cohort study in Norway

**DOI:** 10.2340/aos.v83.40260

**Published:** 2024-05-03

**Authors:** Nivedita Sinha, Harald Johan Hamre, Frauke Musial, Erik L. Werner, Lars Björkman

**Affiliations:** a Centre for International Health, Department of Global Public Health and Primary Care, University of Bergen, Bergen, Norway; b Institute for Applied Epistemology and Medical Methodology, University of Witten/Herdecke, Freiburg, Germany; c Department of Community Medicine, Faculty of Health Sciences, The National Research Center in Complementary and Alternative Medicine (NAFKAM), UiT The Arctic University of Norway, Tromsø, Norway; d Research Unit for General Practice, NORCE Norwegian Research Centre AS, Bergen, Norway; e Department of General Practice, Institute of Health and Society, University of Oslo, Oslo, Norway; f Dental Biomaterials Adverse Reaction Unit, NORCE Norwegian Research Centre AS, Bergen, Norway

**Keywords:** Dental amalgam, prospective cohort study, subjective health complaints, medically unexplained symptoms

## Abstract

**Objective:**

Health complaints attributed to dental amalgam fillings comprise both intraoral and general health complaints. There are data suggesting that patients with medically unexplained physical symptoms (MUPS) attributed to amalgam fillings show improvement in symptoms after removal of all amalgam fillings. However, data indicating changes of specific health complaints are limited. This study evaluated the changes of health complaints after removal of amalgam restorations in patients with health complaints attributed to dental amalgam fillings.

**Method:**

Patients with MUPS attributed to dental amalgam (Amalgam cohort) had all their amalgam fillings removed. The participants indicated an intensity of 11 local and 12 general health complaints on numeric rating scales before the treatment and at follow-up after 1 and 5 years. The comparison groups comprising a group of healthy individuals and a group of patients with MUPS without symptom attribution to dental amalgam did not have their amalgam restorations removed.

**Results:**

In the Amalgam cohort, mean symptom intensity was lower for all 23 health complaints at follow-up at 1 year compared to baseline. Statistically significant changes were observed for specific health complaints with effect sizes between 0.36 and 0.68. At the 5-year follow-up, the intensity of symptoms remained consistently lower compared to before the amalgam removal. In the comparison groups, no significant changes of intensity of symptoms of health complaints were observed.

**Conclusion:**

After removal of all amalgam restorations, both local and general health complaints were reduced. Since blinding of the treatment was not possible, specific and non-specific treatment effects cannot be separated.

## Introduction

Dental amalgam is a combination of liquid mercury and a metal powder referred to as alloy. The alloy consists mainly of silver, tin, and copper. The mixing ratio is 50 wt.% of each [1]. Dental amalgam has been used for restoration of dental caries lesions for more than 150 years as it has excellent durability, low sensitivity towards moisture, and is inexpensive [2]. A significant drawback of dental amalgam is the release of mercury from the fillings [1, 3], which contributes significantly to the daily exposure to inorganic mercury. The daily dose of mercury derived from dental amalgam is estimated to be about 5–9 µg Hg/day in subjects with an ordinary number of amalgam fillings [4]. This makes dental amalgam one of the biggest sources of mercury exposure [5].

The amalgam fillings emit elemental mercury vapor (Hg^0^) that is inhaled and absorbed into the blood stream. About 80% of inhaled mercury vapor is absorbed and retained [6]. Since mercury is continuously released from dental amalgam restorations [7, 8] there is a potential risk for chronic mercury toxicity [1, 3]. Exposure to elemental mercury vapor can have both systemic and local effects [6]. In addition, it has impacts on both the peripheral and the central nervous system [6]. The special affinity of mercury to bind with protein and amino acids [9] and the wide spread occurrence make it to one of the chemicals of major public health concern [10]. However, there are divided views on the risk associated with the use of dental amalgam. Nevertheless, there is a significant concern regarding the potential health effects from mercury vapor when using amalgam as a dental restorative material [11]. Health complaints attributed to the use of dental amalgam are non-specific and heterogeneous [12], which complicates research on the topic. Altogether there is lack of consistent and conclusive evidence to directly correlate amalgam with general adverse health effects.

Localized side effects due to dental amalgam are well-documented. These are mainly related to amalgam tattoos and amalgam-associated oral lichenoid lesions (OLL). OLL are largely associated with delayed hypersensitivity reactions [13]. Removal of the restorations results in disappearance of the lesion in most cases [14], which suggests a causal relationship. Moreover, in some studies, positive correlations have been observed with the use of dental amalgam and general side effects. The general side effects could potentially be related to the release of mercury from dental amalgam [1, 6, 15, 16]. However, the effects from individual behaviors (e.g., bruxism and chewing which is associated with increased release of mercury from dental amalgam restorations [8, 17]) and possible effect modifiers like genetic polymorphisms are not known in detail [16, 18].

Although there are controversies regarding toxicity associated with the use of dental amalgam [19], dental amalgam is still a popular choice in various parts of the world [20], and hence there is concern and worry in the population about the use of dental amalgam [21]. Patients with health complaints attributed to their amalgam fillings have in general several symptoms in common with patients with medically unexplained physical symptoms (MUPS) [22]. The similarity of the health complaints in these two patient groups allows comparison over time.

In 2012 the Norwegian Directorate of Health initiated an experimental treatment project including patients with subjective health complaints attributed to dental amalgam. The project was based on a White paper (*stortingsmelding*) published by the Norwegian Ministry of Health and Care Services [23]. The current paper is based on this project, which is subsequently referred to as ’Bergen Amalgam Trial’ [24]. The Bergen Amalgam Trial was designed as a prospective cohort study with three non-equivalent groups: Amalgam cohort, MUPS cohort, and Healthy cohort. The target population was the Amalgam cohort (patients with MUPS attributed to dental amalgam restorations) who had all amalgam restorations removed and replaced with other dental restorative materials. The MUPS cohort and the Healthy cohort were used as comparison groups. The primary outcome of the project was general health complaints index (GHC-index) 1 year after removal of all amalgam fillings was completed, which was significantly decreased (*p* < 0.001) in the Amalgam cohort [24]. Patients in the comparison cohorts did not have their amalgam fillings removed and there was no significant change in the GHC-index at follow-up in these cohorts. Changes in the specific health complaints were not presented in the previous publication [24], in which a comparative analysis of GHC change in the three groups was presented. The main objective of the current paper is to present a descriptive analysis characterizing changes of specific health complaints in the Amalgam cohort after removal of all amalgam restorations over time periods of 1 and 5 years after amalgam removal.

## Material and methods

Data were collected in the Bergen Amalgam Trial, which was organized by the Dental Biomaterials Adverse Reaction Unit in Bergen, Norway, on behalf of the Norwegian Directorate of Health. In this paper, data from the project are presented to describe the change in observed health complaints after removal of the amalgam restorations.

### Participants

**Figure 1:**
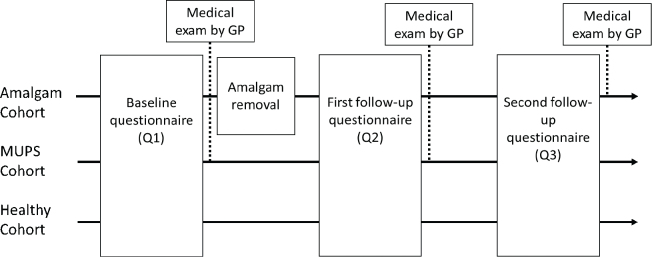
Timeline of the Bergen Amalgam Trial. Three cohorts were included using a prospective cohort study design. Participants in the Amalgam cohort and MUPS cohort were examined by their GP (General Practitioner) at baseline (Q1) and at the first follow up (Q2). Participants in the Amalgam cohort were also examined by their GP at the second follow-up (Q3).

Participants in the Amalgam cohort sent an application to the study office for participation in the project. Participants in the MUPS cohort were recruited via their family physician/general practitioner, and participants in the Healthy cohort were recruited mainly via dentists participating in the project. A simplified timeline of the Bergen Amalgam Trial is given in Figure 1. Recruitment period for the participants was from March 2013 to December 2015.

### Inclusion and exclusion criteria

The inclusion criteria included age between 20 and 70 years, ability to comply with the project, and permanent residence in Norway. A complete description of inclusion and exclusion criteria for all three cohorts is given in Table 1. Details regarding recruitment and medical and dental examinations are given elsewhere [24].

**Table 1:** Inclusion and exclusion criteria for all three cohorts (source: reference 24).

	Cohort		
	Amalgam	MUPS	Healthy
**Inclusion criteria**			
Age between 20 and 70 years	x	x	x
Permanent residents in Norway	x	x	x
Able to comply with the protocol	x	x	x
Health complaints attributed (by the patient) to dental amalgam restorations	x		
No attribution to amalgam and no explicit wish to remove amalgam		x	
≥3 months duration of the health complaints attributed (by the patient) to amalgam restorations	x		
≥3 months duration of unspecific health complaints		x	
Presence of at least one amalgam filling	x		
Wish to have all amalgam fillings removed	x		
Examination by patient’s physician and dentist according to guidelines from the Norwegian Directorate of Health	x		
Diagnosed diseases adequately treated	x	x	
Patient’s general practitioner/family physician and dentist assess that the general and dental health of the patient most likely not will deteriorate due to participation in the project	x		
Patient’s dentist assess that there are no major risks for complications following amalgam removal (e.g., need for root canal treatments or extractions)	x		
Subjective symptoms without corresponding objective findings after medical examination(s), including symptoms not explained by patient’s diagnoses	x	x	
Moderate or severe functional impairment (physician-assessed)	x	x	
Subjectively healthy (self-assessed)			x
No medication (intake of vitamins and minerals allowed)			x
**Exclusion criteria**			
Pregnancy (or planned pregnancy) and lactation	x	x	x
Life threatening disease	x	x	n.a.
Patients with ongoing cancers, severe cardiopulmonary, neurological, or psychiatric diseases (assessed by the GP)	x	x	n.a.
Organic cause of all complaints (according to checklist, see text)	x	x	n.a.

n.a.: not applicable.

### Data collection

At baseline, the first questionnaire (Q1) was sent to all three cohorts after written informed consent was obtained. This was followed by removal of amalgam restorations in the Amalgam cohort. A follow-up questionnaire (Q2) was sent by mail to the patients in the Amalgam cohort 1 year after removal of the last amalgam restoration. Q2 was sent to the comparison groups 2 years after the completion of the baseline questionnaire. Four years after completion of Q2, the second follow-up questionnaire (Q3) was distributed to the cohorts.

### Primary outcome

The primary outcome of the Bergen Amalgam Trial was the GHC-index at 12-month follow-up after amalgam removal was completed. The GHC-index is the sum score of 12 items, scored by numeric rating scales from 0 (no symptoms) to 10 (worst imaginable symptoms) (Table 2). The GHC-index is a valid instrument with adequate sensitivity to change over time for assessing symptom intensity in MUPS patients with health complaints attributed to amalgam fillings and undergoing amalgam removal [25]. In addition to the 12 GHC-items, local (intraoral and extra oral) complaints were included for the Amalgam cohort and for the Healthy cohort. The local orofacial complaints were further divided into six intraoral and five extra oral health complaints (Table 2).

**Table 2:** Local and general health complaints included in the questionnaires. Local (orofacial) health complaints were further divided into 6 intraoral and 5 extraoral items. General health complaints included 12 items.

Health complaints		
Local (orofacial) health complaints		General health complaints *
Intraoral	Extraoral	
Intraoral burning sensation	Facial burning sensation	Pain from muscles and joints
Intraoral pain/tenderness	Facial pain/tenderness	Gastrointestinal symptoms
Taste disturbances	Facial stiffness/paresthesia	Cardiovascular symptoms
Intraoral stiffness/paresthesia	Facial skin problems	General skin problems
Dry mouth	Pain from temporomandibular joints	Visual disturbances
Increased salivation/mucus		Symptoms from ear/nose/throat
		Fatigue
		Dizziness
		Headache
		Memory problems
		Difficult to concentrate
		Anxiety/depression

* The GHC-index is the sumscore of the 12 general health complaints included.

### Secondary outcome

The Munich Amalgam Scale was included in the questionnaires distributed to the Amalgam cohort [26]. The scale includes 50 items (Supplemental Table S1), and each item was scored 0 (not at all), 1 (a little), 2 (quite a lot), or 3 (very much).

### Statistical analysis

Data from the numeric rating scales and the Munich Amalgam Scale were treated as interval data assuming equal spacing between adjacent values. We tested the hypothesis (H_0_) that there was no change in intensity of the separate local and general symptoms in the three cohorts from baseline (Q1) to the first follow-up (Q2) and the second follow-up (Q3), respectively. The hypothesis was tested separately for each cohort for each of the 23 items. Data were analyzed by linear mixed models using restricted maximum-likelihood (REML) taking the longitudinal design into consideration. The distribution of residuals was checked for normality. When residuals were skewed, Friedman’s test was used to calculate significance for change over time within the group. Effect size (ES) was estimated by calculating the ‘standardized response mean’ (SRM) by dividing the mean change score with the standard deviation of the change score. Values of 0.20 represent a small response, values of 0.50 represent a medium response, and values of 0.80 represent a large response [27].

The statistical software IBM-SPSS (IBM Corp. Released 2020. IBM SPSS Statistics for Windows, Version 27.0. Armonk, NY: IBM Corp) and STATA (StataCorp. 2021. Stata Statistical Software: Release 17. College Station, TX: StataCorp LLC) were used for the calculations. *P*-values less than 0.05 were considered statistically significant. No adjustment for multiple hypothesis testing was made [28].

### Ethical considerations

The project was approved by the local research ethics committee (REK2012/331; https://rekportalen.no), and registered at ClinicalTrials.gov(https://clinicaltrials.gov/ct2/show/NCT01682278). All participants signed an informed consent form.

## Results

**Figure 2:**
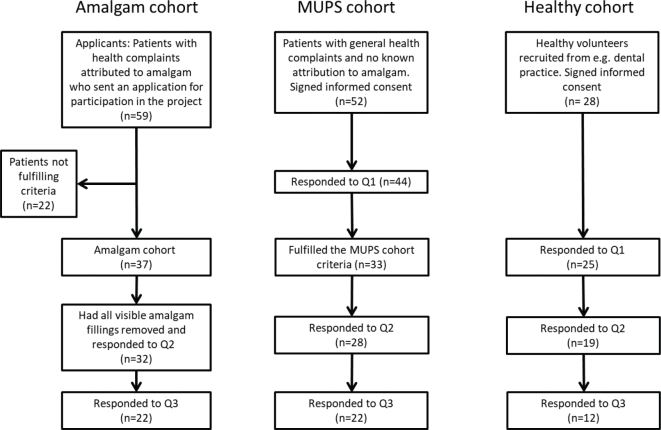
Flowchart of the Bergen Amalgam Trial.

A total of 59 patients with health complaints attributed to dental amalgam sent an application for participation in the project (Figure 2). After consideration of inclusion and exclusion criteria, 37 participants were included in the Amalgam cohort. A total of 32 participants had all amalgam fillings removed and responded to Q2.

Fifty-two patients were recruited to the MUPS cohort and signed an informed consent. Among these, 44 participants responded to the baseline questionnaire. In total, 33 patients fulfilled the MUPS cohort criteria, and of these 28 responded to both Q1 and Q2.

The third cohort consisted of healthy volunteers and was mainly recruited from dental practice. Among 28 participants who signed an informed consent, 25 responded to Q1 and six of them did not respond to Q2. Thus, 19 were available for the analysis of change scores between baseline and Q2 (Table 3).

**Table 3:** Baseline characteristics for the Amalgam cohort (*n* = 32), MUPS cohort (*n* = 28) and the Healthy cohort (*n* = 19).

		Cohort		
		Amalgam (*n* = 32)	MUPS (*n* = 28)	Healthy (*n* = 19)
Gender	Male	13 (40.6%)	4 (14.3%)	6 (31.6%)
	Women	19 (59.4%)	24 (85.7%)	13 (68.4%)
Age (years); mean (SD)		52.1 (7.5)	49.9 (10.3)	46.9 (13.1)
Education; n (%)	Primary and lower secondary school	3 (9.4%)	3 (10.7%)	0 (0.0%)
	Upper secondary school, vocational program	9 (28.1%)	7 (25.0%)	1 (5.3%)
	Upper secondary school, general program	2 (6.3%)	7 (25.0%)	4 (21.1%)
	Higher education (less than 4 years)	1 (34.4%)	9 (32.1%)	6 (31.6%)
	Higher education (4 years or more)	7 (21.9%)	2 (7.1%)	8 (42.1%)
Civil status; n (%)	Married	24 (75.0%)	20 (71.4%)	9 (47.4%)
	Cohabitation	2 (6.3%)	3 (10.7%)	4 (21.1%)
	Single	2 (6.3%)	4 (14.3%)	4 (21.1%)
	Divorced	4 (12.5%)	1 (3.6%)	2 (10.5%)
Smoking habits; n (%)	No, never ever	13 (40.6%)	15 (55.6%)	11 (57.9%)
	No, stopped less than 1 year ago	1 (3.1%)	1 (3.7%)	0 (0.0%)
	No, stopped more than 1 year ago	14 (43.8%)	6 (22.2%)	5 (26.3%)
	Yes, but not daily	0 (0.0%)	3 (11.1%)	2 (10.5%)
	Yes, daily	4 (12.5%)	2 (7.4%)	1 (5.3%)
GHC; mean (SD)		43.3 (17.8)	36.6 (14.2)	10.0 (15.0)

The second follow-up questionnaire (Q3) was completed by 22 participants in the Amalgam cohort, 22 participants in the MUPS cohort, and by 12 participants in the Healthy cohort.

## Amalgam cohort: Local and general health complaints

### Symptom reduction

**Figure 3:**
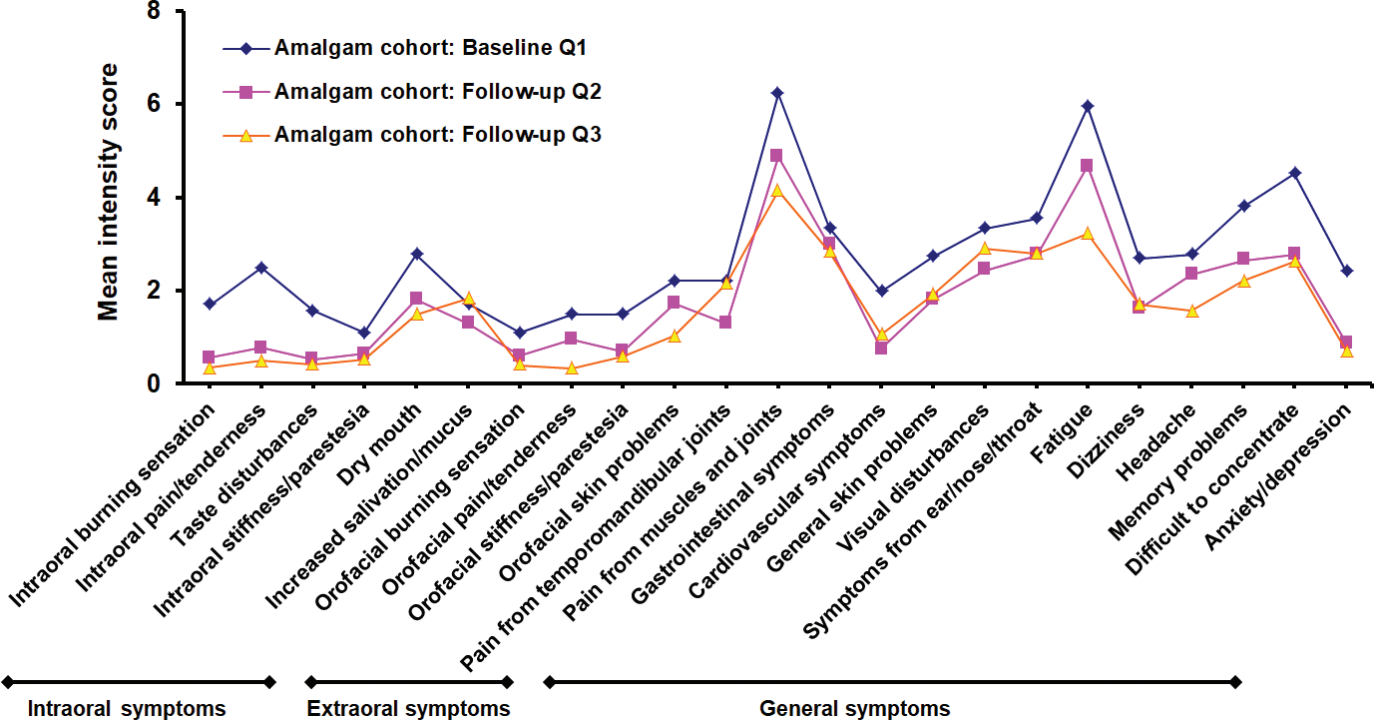
Mean intensity score for local and general health complaints at baseline (Q1) and at first (Q2) and second (Q3) follow-up for the Amalgam cohort.

**Figure 4:**
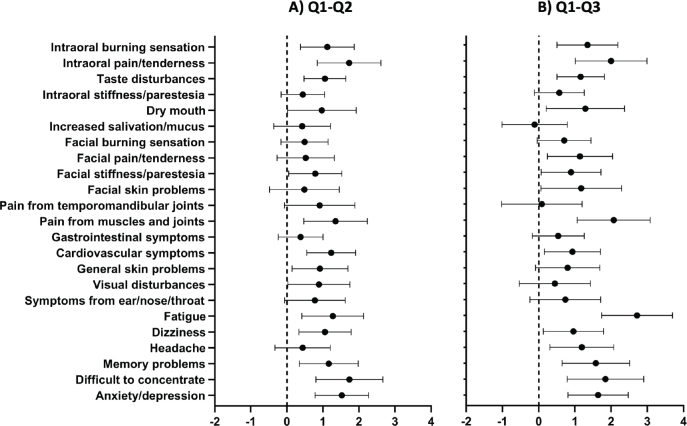
Mean difference and 95% confidence interval in intensity of local and general health complaints. A) Baseline – 1-year follow-up (Q1–Q2) and B) Baseline – 5-year follow-up (Q1–Q3) in the Amalgam cohort. Positive value indicates improvement of symptoms after removal of amalgam restorations. The dashed line indicates zero change.

In the Amalgam cohort the mean intensity of each of all 23 items was reduced at the first follow-up (Q2) compared with the baseline (Q1) (Figure 3). Fourteen of the symptoms were significantly reduced (Table 4). At the second follow-up (Q3), reduction in mean intensity was observed for all but one item compared with baseline (Q1) (Figure 4).

**Table 4:** Mean intensity scores and standard errors (SE) for local and general health complaints at baseline (Q1) and the first (Q2) and second (Q3) follow-ups for the Amalgam cohort (*n* = 32). P-values calculated by linear mixed models for the test of change from baseline are given in addition (H_0_: Change score = 0).

	Q1		Q2			Q3		
	Mean	SE*	Mean	SE*	P	Mean	SE*	P
*Local symptoms*								
Intraoral burning sensation	1.7	0.3	0.6	0.3	0.003	0.3	0.3	0.002
Intraoral pain/tenderness	2.5	0.3	0.8	0.3	<0.001	0.5	0.3	<0.001
Taste disturbances	1.6	0.2	0.5	0.2	<0.001	0.4	0.2	0.001
Intraoral stiffness/paresthesia	1.1	0.2	0.6	0.2	0.148	0.5	0.2	0.104
Dry mouth	2.8	0.4	1.8	0.4	0.046	1.5	0.4	0.019
Increased salivation/mucus	1.7	0.4	1.3	0.4	0.289	1.8	0.4	0.812
Orofacial burning sensation	1.1	0.2	0.6	0.2	0.139	0.4	0.3	0.062
Orofacial pain/tenderness	1.5	0.3	1.0	0.3	0.194	0.3	0.3	0.013
Orofacial stiffness/paresthesia	1.5	0.3	0.7	0.3	0.035	0.6	0.3	0.034
Orofacial skin problems	2.2	0.4	1.7	0.4	0.323	1.0	0.4	0.039
Pain from temporomandibular joints	2.2	0.4	1.3	0.4	0.067	2.2	0.5	0.874
*General symptoms*								
Pain from muscles and joints	6.2	0.5	4.9	0.5	0.003	4.2	0.5	<0.001
Gastrointestinal symptoms	3.4	0.4	3.0	0.4	0.226	2.8	0.5	0.140
Cardiovascular symptoms	2.0	0.3	0.8	0.3	<0.001	1.1	0.3	0.018
General skin problems	2.7	0.4	1.8	0.4	0.020	1.9	0.4	0.076
Visual disturbances	3.3	0.4	2.4	0.4	0.042	2.9	0.4	0.372
Symptoms from ear/nose/throat	3.5	0.4	2.8	0.4	0.069	2.8	0.4	0.140
Fatigue	5.9	0.5	4.7	0.5	0.003	3.2	0.5	<0.001
Dizziness	2.7	0.4	1.6	0.4	0.004	1.7	0.4	0.024
Headache	2.8	0.5	2.3	0.5	0.261	1.6	0.5	0.008
Memory problems	3.8	0.5	2.6	0.5	0.005	2.2	0.5	0.001
Difficult to concentrate	4.5	0.4	2.8	0.4	<0.001	2.6	0.5	0.001
Anxiety/depression	2.4	0.4	0.9	0.4	<0.001	0.7	0.4	<0.001

* Delta method.

### Consistent significant changes

Five local symptoms were significantly decreased from baseline at both follow-ups: ‘intraoral burning sensation’, ‘intraoral pain/ tenderness’, ‘taste disturbance’, ‘dry mouth’, and ‘facial stiffness/paresthesia’ (Table 4 and Figure 4). Seven of the GHC were consistently changed: ‘pain from muscles and joints’, ‘cardiovascular symptoms’, ‘fatigue’, ‘dizziness’ ‘memory problems’, ‘difficulty to concentrate’, and ‘anxiety/depression’ (Table 4 and Figure 4).

### Effect sizes and mean change scores

**Figure 5:**
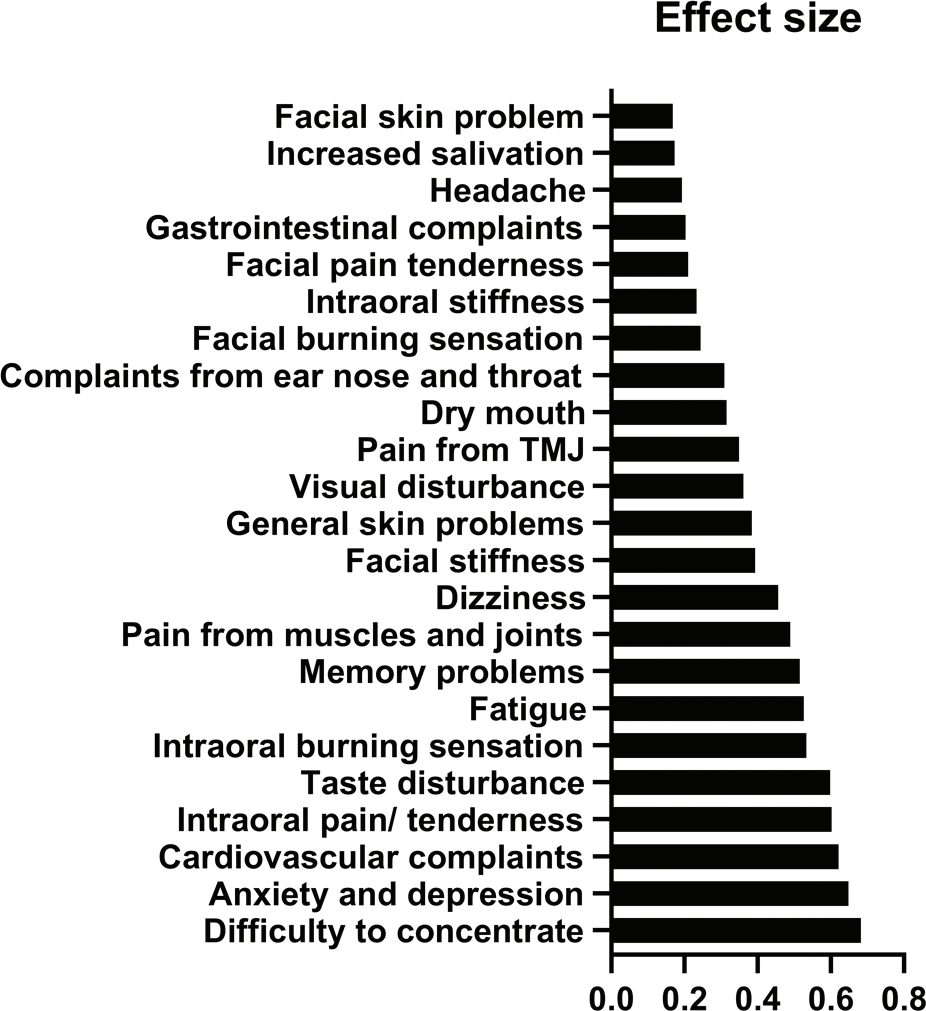
Effect size (ES) (standardized response mean) for all 23 symptoms after amalgam restoration removal in first follow-up period (Q1–Q2).

The ESs, estimated by the SRM, for the intraoral symptoms ‘intraoral burning sensation’, ‘intraoral pain/tenderness’, and ‘taste disturbance’, were around 0.6 representing a medium response. ESs were small for extra oral health complaints at the first follow-up (Q2) (Figure 5). The mean change score for intraoral complaints was highest for pain/tenderness followed by burning sensation, taste disturbance, dry mouth, intraoral stiffness, and increased salivation at first follow-up (Q2) (Figure 4). Medium to large ESs (between 0.5 and 0.8) were observed for the GHC ‘cardiovascular complaints’, ‘fatigue’, ‘memory problems’, ‘difficulty to concentrate’, and ‘anxiety/depression’ (Figure 5). Mean change score at the first follow-up was highest for ‘difficulty to concentrate’ followed by ‘anxiety/depression’, and ‘pain from muscles and joints’ (Figure 4).

### Amalgam cohort: Munich Amalgam Scale

Patients in the Amalgam cohort also responded to the Munich Amalgam Scale. The highest ESs (estimated by the SRM) were found for ‘metallic taste’ (0.93), ‘stress at the job’ (0.83), ‘skin rash’ (0.57), ‘sensitivity to cold and wind’ (0.54), ‘worries – restlessness’ (0.53), and ‘irritability’ (0.52); and significant reduction of intensity of symptoms was shown (Supplemental Table S1). Moderate ESs (around 0.4) were found for items ‘feeling of walking next to oneself’, ‘arrythmia’, ‘diarrhea’, ‘increased urge to toilet’, ‘indecision’, ‘abdominal pain’, ‘lack of concentration’, ‘fluctuating mood’, ‘anxiety’, and ‘burning sensation in tongue’, all being statistically significant (Supplemental Table S1).

### MUPS cohort

For the MUPS cohort no significant changes were observed for GHC (Supplemental Table S2). Data for local complaints were not available for the MUPS cohort.

### Healthy cohort

For the Healthy cohort there were no significant changes for local and GHC (Supplemental Table S3).

### Effects associated with age and gender

Explorative analyses of the Amalgam cohort including age and gender in the mixed effects model indicated significant effects from both age and gender for the item ‘pain from muscles and joints’. Women had, at an average, a higher overall intensity of ‘pain from muscles and joints’ (mean 1.8; 95% confidence interval [CI] from 0.4 to 3.4, *p* = 0.016). An increase of 10 years of age was associated with lower intensity of ‘pain from muscles and joints’ (mean 1.3; 95% CI from 0.2 to 2.3; *p* = 0.014). Significant effects from age, but not for gender, were observed for the symptoms ‘fatigue’, ‘intraoral stiffness/paresthesia’, and ‘pain from temporomandibular joints’. An increase of 10 years of age was associated with a lower ‘fatigue’ symptom intensity (1.5; 95% CI from 0.4 to 2.6; *p* = 0.008), lower ‘intraoral stiffness/paresthesia’ (0.6; 95% CI from 0.2 to 1.0; *p* = 0.005), and lower ‘pain from temporomandibular joints’ (1.3; 95% CI from 0.4 to 2.2; *p* = 0.005).

## Discussion

### The main finding – mean intensity of all symptoms decreased

The main finding of this study was that all mean values for both local complaints and GHC were reduced in the Amalgam cohort 1 year after removal of amalgam restorations (Figures 3 and 4). The largest reductions (ES about 0.6) were observed for intraoral pain/tenderness, taste disturbance, cardiovascular complaints, difficulty concentrating, and anxiety/depression (Figure 5). In the comparison cohorts, there were no significant changes over time, and the mean ESs were around 0. To our knowledge this is the first study to explore the specific types of symptoms that change after removal of amalgam restorations in patients with MUPS who had all their amalgam restorations replaced with other restorative materials.

### Consistent results at both follow-ups

The reduction of complaint intensity in the Amalgam cohort was still seen at the second follow-up 5 years after amalgam removal (Table 4). In the comparison cohorts, mean values at the second follow-up were not significantly different from baseline values (Supplemental Tables S2 and S3).

### Consistencies between items

The Munich Amalgam Scale is based on scores from 0 to 3 (not at all – a little – quite a lot – very much) for each of the 50 items, while the 23 items in the Health Complaints Scale are based on scores from numeric rating scales from 0 to 10. Seven items were partly overlapping between the Munich Amalgam Scale (Fatigue, Lack of concentration, Headache, Dry mouth, Metallic taste, Visual disorders, Skin rash; see Supplemental Table S1) and the Health Complaints Scale (Fatigue, Difficult to concentrate, Headache, Dry mouth, Taste disturbances, Visual disturbances, General skin problems; see Table 2). The ESs found for the seven overlapping items in MAS were plotted against the ESs found for the corresponding items included in the Health Complaints Scale. The correlation coefficient was 0.659 (*p* = 0.108), and there was an acceptable agreement between the items from the scales. The scale differences (coded 0–3 in MAS and coded 0–10 in the Health Complaints Scale) could probably explain the far from perfect correlation.

### Effect sizes and clinical relevance

Effect size helps in understanding the magnitude of difference found and is effective in determining the clinical meaningfulness of the finding [29]. In this study, it is noteworthy that ESs were medium to large for several items suggesting clinically meaningful results along with statistically significant results.

### Other studies

The same 23 items (the 12 general complaints and the 11 local complaints) were used in a previous study which showed reduction of health complaints for 20 out of 23 health complaints and statistically significant reductions were observed at follow-up 3 years after the removal of amalgam fillings for ‘taste disturbance’, ‘pain from muscles and joints’, ‘symptoms from ear, nose and throat’, and ‘difficulty concentrating’. In our study, reduction in symptoms was observed for all 23 health complaints and significant results were obtained for 14 out of 23 health complaints at the first follow-up (Q2) and 15 out of 23 health complaints at the second follow-up (Q3). This disparity in intensity of symptoms could be due to the difference in sample size and follow-up period; the present study was based on a larger sample size compared with the previous study and the follow-up time in the two studies was different [30].

The results of this study correspond well with other studies, where marked reduction of health complaints attributed to dental amalgam were observed after replacement with other restorative materials [12, 31, 32]. In some studies, associations between symptoms and markers of mercury exposure were reported suggesting a possible causal effect from mercury exposure [15, 33]. Similarly, a study conducted by Lindh et al. supported the hypothesis that metal exposure from dental amalgam can cause ill health in the susceptible population [34]. More than 70% of the responders who were given replacement of the metal restoration and proper oxidative therapy showed improvement of the symptoms [34].

### Strengths and limitations

This study is best defined as a prospective cohort study with three non-equivalent groups, since the intervention was not assigned by us (the researchers) or by the study office, and the cohorts were only observed over time. Alternatively, the study design could be described as a quasi-experimental study or a controlled before-and-after study, but in these designs the researcher usually controls the allocation to the groups. The prospective cohort study design allowed us to follow the groups over time and collect data both at baseline and follow-ups.

In the Amalgam cohort, 22 of 32 participants could be followed up at Q3. The use of linear mixed models that calculate estimates taking non-response into consideration, minimizes the potential risk of selection bias due to loss of follow-up. Even though the sample size was relatively small, there was adequate power to show changes in intensity of several symptoms after removal of amalgam restorations [24]. Results are mainly based on ES and change score calculation to illustrate changes before and after treatment which is clinically meaningful.

To minimize possible information bias (like desirability bias) and a potential Hawthorne effect, there were no physical meetings between participants and investigators and responses were based only on postal questionnaires. To minimize recall bias, measurement of response was done at three specific times during the study, first at baseline and then at a follow-up 1 and 5 years after removal of the amalgam restorations. Thus, we measured point prevalence of intensity of symptoms without delay in time.

Since the study could not be blinded and both participants and investigators were aware of the type of intervention given, effects from placebo cannot be ignored. However, the changes of complaint intensity are the sum of both specific treatment effect (reduction of exposure) and possible effects from placebo, expectations, general care, etc. The aim of the study was not to quantify the effect from reduced exposure to amalgam, but to quantify possible changes of health after removal of amalgam restorations. In addition, randomization of participants was not feasible due to the nature of the study (scientific evaluation of a treatment project initiated by a national health authority, with high risk of drop out of patients randomized to a control group with no amalgam removal).

We used items from the GHC index as a tool to measure change of symptom intensity before and after removal of amalgam restorations in patients with MUPS. It has been shown that both the GHC index and the Munich amalgam scale are valid and responsive instruments for assessment of symptom load in MUPS patients attributing their health complaints to amalgam fillings and undergoing amalgam removal [25].

Health complaint intensity was measured by numeric rating scales. The measure was based on subjective interpretation of symptoms by patients and was self-reported, and there were no uniform objective assessment criteria. In addition, there may be chances that reduction in intensity of symptoms follows a natural course of recovery. The design allowed us to control for natural recovery and usual care by using the MUPS cohort with symptoms similar to the comparison group. No significant change in intensity of symptoms was observed for the MUPS cohort at the follow-ups. In addition, five items regarding local complaints (‘intraoral burning sensation’, ‘intraoral pain and tenderness’, ‘taste disturbance’, ‘dry mouth’, and ‘facial stiffness’) and seven items regarding general complaints (‘pain from muscles and joints’, ‘cardiovascular complaints’, ‘fatigue’, ‘dizziness’, ‘memory problems’, ‘difficulty to concentrate’, and ‘anxiety/ depression’) showed significant change both at 1 and 5 years follow-up which may reduce the probability of placebo as the only cause of symptom reduction (Table 4).

### External validity

Due to the use of inclusion and exclusion criteria, generalizability is limited to patients having MUPS and attributing symptoms to dental amalgam and those who fulfilled the inclusion and exclusion criteria. The initial screening of patients for eligibility in the study based on their medical condition was done by their local physician and dentists, and this could result in further limitations of generalizability. Thus, the sample population is not representative for the general Norwegian population.

### Could the reduction of health complaints be caused by reduced exposure to mercury?

In the Amalgam cohort, both intensity of GHC and concentration of inorganic mercury in the serum were significantly decreased after amalgam removal [35]. Experiences from occupational exposure to higher levels of mercury vapor than those associated with exposure to dental amalgam have indicated that symptoms caused by exposure to mercury vapor are characterized as a non-specific, asthenic-vegetative syndrome involving symptoms such as weakness, fatigue, anorexia, loss of weight, and disturbance of gastrointestinal functions, sometimes called ‘micromercurialism’ [6]. Anxiety/depression, fatigue, memory problems, and visual disturbance have also been associated with long-term exposure to high doses of mercury [36]. Symptoms are more prevalent in exposed groups than control groups with lower exposure [6]. A lack of association on the individual level between dose and effects is well-known and it is proposed that this is partly due to individual variability in genetically determined sensitivity to mercury [6]. Thus, it is not possible to exclude that the reduction of symptoms was caused by the reduced exposure even though there was no statistically significant association between the reduction of symptoms and the reduction of inorganic mercury in serum [35].

### Are mercury symptoms reversible?

After a slight poisoning by mercury vapor, symptoms of the poisoning are reduced and could disappear when exposure has ceased [6]. After overt poisoning by high levels of mercury vapor, some severe cases with long-term exposure could have persistent symptoms and effects even after ceased exposure [37, 38]. Thus, it could be expected that symptoms of a slight poisoning (‘micromercurialism’) could disappear when the exposure has ceased [6].

### Causality, association, genetic polymorphism, and mercury toxicity

In this study reduction of the intensity of symptoms after removal of amalgam restorations was observed, which suggests a possible dose–response relationship. The finding of the current study suggests an association between removal of amalgam restorations and improvement of subjective health complaints in the amalgam cohort. However, for conclusions about causality there is generally a need for higher levels of evidence and, in addition, support from laboratory-based objective findings. Moreover, to support the findings from the present study additional data from studies with designs providing higher levels of evidence, like randomized controlled trials (preferably blinded), would be useful. One of the practical challenges is the design of a randomized controlled trial considering the ethical aspect and the obvious challenge of implementing blinded clinical procedures (amalgam removal) further questions the possibility of producing studies with higher level of evidence. Thus, more studies are warranted to determine the underlying susceptibility of individuals towards mercury toxicity, considering individual genetic differences within the population.

Novel studies could test a causal relationship based on the concepts (‘as a rule of thumb’) such as strength, consistency, specificity, temporality, biological gradients, plausibility, coherence, experiment, and analogy as suggested by Hill [39] for establishing causal relationship between exposure (interrupted exposure to amalgam) and outcome (improvement of health complaints) [40]. In summary, studies on changes of health complaints after removal of amalgam restorations show strength (medium to large ES for GHC [31, 32, 41]), consistency (published studies show similar results [15, 32, 33, 41, 42]), specificity (Hg causes symptoms from mainly CNS [6]), temporality (effect after treatment), dose–response (see [15, 42, 43]), plausibility (mercury is toxic [6]), coherence (mercury from dental amalgam is found in the brain and other organs [44]), experiment (removal of a potentially harmful exposure reduces symptoms [15, 32, 33, 41, 42]), and analogy (other toxic metals causes toxic effects as well). Within the limitations of these criteria and applying reservations and exceptions [40], a causal relationship cannot, however, be disregarded.

## Conclusion

This study supports the hypothesis that reduction of intensity of symptoms, which could be caused by mercury, occurs after removal of amalgam restorations in patients with MUPS attributed to amalgam. Since the study was not blinded, there is a possibility of both specific treatment effects (due to reduced exposure to dental amalgam) and non-specific treatment effects (e.g., placebo based on expectations and general care given to patients).

## Supplementary Material

Health complaints before and at one and five years after removal of dental amalgam restorations – data from a prospective cohort study in Norway

## Data Availability

The data contain potentially identifying and sensitive patient information and are not available due to personal data protection regulations. The restrictions are imposed by NORCE’s Administrative Support for Research and the Data Protection Officer at NORCE.

## References

[1] Shen C. Dental amalgams. In: Anusavice KJ, Shen C, Rawls HR, editors. Phillips’ science of dental materials. 12th ed. St. Louis, MO: Elsevier/Saunders; 2013. p. 340–63.

[2] SCENIHR (Scientific Committee on Emerging and Newly-Identified Health Risks). Opinion on the safety of dental amalgam and alternative dental restoration materials for patients and users (update), 29 April, 2015. Luxembourg: European Commission, DG Health and Food Safety; 2015.

[3] Bates MN. Mercury amalgam dental fillings: an epidemiologic assessment. Int J Hyg Environ Health. 2006 Jul;209(4):309–16. 10.1016/j.ijheh.2005.11.006 16448848

[4] Sandborgh-Englund G, Elinder CG, Johanson G, et al. The absorption, blood levels, and excretion of mercury after a single dose of mercury vapor in humans. Toxicol Appl Pharmacol. 1998;150(1):146–53. 10.1006/taap.1998.8400 9630463

[5] Risher JF, World Health Organization & International Programme on Chemical Safety. Elemental mercury and inorganic mercury compounds: human health aspects. World Health Organization; 2003. [cited 2024 March 12] Available from: https://apps.who.int/iris/handle/10665/42607

[6] Berlin M, Zalups RK, Fowler BA. Mercury. In: Nordberg GF, Fowler BA, Nordberg M, editors. Handbook on the toxicology of metals. Amsterdam, Netherlands: Elsevier; 2014. p. 1013–75.

[7] Svare CW, Peterson LC, Reinhardt JW, et al. The effect of dental amalgams on mercury levels in expired air. J Dent Res. 1981;60(9):1668–71. 10.1177/00220345810600090601 6943160

[8] Björkman L, Lind B. Factors influencing mercury evaporation rate from dental amalgam fillings. Scand J Dent Res. 1992;100(6):354–60. 10.1111/j.1600-0722.1992.tb01086.x 1465570

[9] Clarkson TW, Magos L. The toxicology of mercury and its chemical compounds. Crit Rev Toxicol. 2006 Sep;36(8):609–62. 10.1080/10408440600845619 16973445

[10] World Health Organization. 10 chemicals of public health concern. World Health Organization; 2020 [cited 2023 Apr 27]. Available from: https://www.who.int/news-room/photo-story/photo-story-detail/10-chemicals-of-public-health-concern

[11] Brownawell AM, Berent S, Brent RL, et al. The potential adverse health effects of dental amalgam. Toxicol Rev. 2005;24(1):1–10. 10.2165/00139709-200524010-00001 16042501

[12] Sjursen TT, Binder P-E, Lygre GB, et al. How unexplained health complaints were attributed to dental amalgam. Nordic Psychol. 2014;66(3):216–29. 10.1080/19012276.2014.964958

[13] McCullough MJ, Tyas MJ. Local adverse effects of amalgam restorations. Int Dent J. 2008 Feb;58(1):3–9. 10.1111/j.1875-595X.2008.tb00170.x 18350847

[14] Issa Y, Duxbury AJ, Macfarlane TV, et al. Oral lichenoid lesions related to dental restorative materials. Br Dent J. 2005 Mar 26;198(6):361–6; disussion 549; quiz 372. 10.1038/sj.bdj.4812176 15789104

[15] Weidenhammer W, Bornschein S, Zilker T, et al. Predictors of treatment outcomes after removal of amalgam fillings: associations between subjective symptoms, psychometric variables and mercury levels. Community Dent Oral Epidemiol. 2010 Apr;38(2):180–9. 10.1111/j.1600-0528.2009.00523.x 20074291

[16] Berlin M. Mercury in dental amalgam: a risk analysis. Neurotoxicology. 2020 Dec;81:382–6. 10.1016/j.neuro.2020.09.034 35623360

[17] Richardson GM. Assessment of mercury exposure and risks from dental amalgam. Final report. Medical Devices Bureau, Environmental Health Directorate. Health Canada, Ottawa; 1995.

[18] Andreoli V, Sprovieri F. Genetic aspects of susceptibility to mercury toxicity: an overview. Int J Environ Res Public Health. 2017 Jan 18; 14(1):93. 10.3390/ijerph14010093 28106810 PMC5295343

[19] Rathore M, Singh A, Pant VA. The dental amalgam toxicity fear: a myth or actuality. Toxicol Int. 2012 May;19(2):81–8. 10.4103/0971-6580.97191 22778502 PMC3388771

[20] Rasines Alcaraz MG, Veitz-Keenan A, Sahrmann P, et al. Direct composite resin fillings versus amalgam fillings for permanent or adult posterior teeth. Cochrane Database Syst Rev. 2014 Mar 31(3):Cd005620. 10.1002/14651858.CD005620.pub2 24683067

[21] Spencer AJ. Dental amalgam and mercury in dentistry. Aust Dent J. 2000 Dec;45(4):224–34. 10.1111/j.1834-7819.2000.tb00256.x 11225523

[22] Jackson JL, George S, Hinchey S. Medically unexplained physical symptoms. J Gen Intern Med. 2009 Apr;24(4):540–2. 10.1007/s11606-009-0932-x 19255810 PMC2659163

[23] Norwegian Ministry of Health and Care Services. Tilgjengelighet, kompetanse og sosial utjevning. Framtidas tannhelsetjenester. Oslo: Helse-og omsorgsdepartementet; 2006.

[24] Björkman L, Musial F, Alraek T, et al. Removal of dental amalgam restorations in patients with health complaints attributed to amalgam: a prospective cohort study. J Oral Rehabil. 2020 Nov;47(11):1422–34. 10.1111/joor.13080 32810306

[25] Lamu AN, Robberstad B, Hamre HJ, et al. Validity and responsiveness of GHC-index in patients with amalgam-attributed health complaints. Acta Odontol Scand. 2021 Oct 15:1–8. 10.1080/00016357.2021.1989032 34651557

[26] Melchart D, Wuhr E, Weidenhammer W, et al. A multicenter survey of amalgam fillings and subjective complaints in non-selected patients in the dental practice. Eur J Oral Sci. 1998 Jun;106(3):770–7. 10.1046/j.0909-8836.1998.eos106303.x 9672099

[27] Sawilowsky SS. New effect size rules of thumb. J Modern Applied Statistical Methods. 2009;8(2 Article 26):597–9. 10.22237/jmasm/1257035100

[28] Rothman KJ. No adjustments are needed for multiple comparisons. Epidemiology. 1990 Jan;1(1):43–6. 10.1097/00001648-199001000-00010 2081237

[29] Sullivan GM, Feinn R. Using effect size-or why the P value is not enough. J Grad Med Educ. 2012 Sep;4(3):279–82. 10.4300/JGME-D-12-00156.1 23997866 PMC3444174

[30] Lygre GB, Sjursen TT, Svahn J, et al. Characterization of health complaints before and after removal of amalgam fillings – 3-year follow-up. Acta Odontol Scand. 2013 May-Jul;71(3–4):560–9. 10.3109/00016357.2012.697577 22746255

[31] Melchart D, Vogt S, Kohler W, et al. Treatment of health complaints attributed to amalgam. J Dent Res. 2008 Apr;87(4):349–53. 10.1177/154405910808700410 18362317

[32] Nerdrum P, Malt UF, Hoglend P, et al. A 7-year prospective quasi-experimental study of the effects of removing dental amalgam in 76 self-referred patients compared with 146 controls. J Psychosom Res. 2004 Jul;57(1):103–11. 10.1016/S0022-3999(03)00542-7 15256302

[33] Zwicker JD, Dutton DJ, Emery JC. Longitudinal analysis of the association between removal of dental amalgam, urine mercury and 14 self-reported health symptoms. Environ Health. 2014;13:95. 10.1186/1476-069X-13-95 25404430 PMC4273453

[34] Lindh U, Hudecek R, Danersund A, et al. Removal of dental amalgam and other metal alloys supported by antioxidant therapy alleviates symptoms and improves quality of life in patients with amalgam-associated ill health. Neuro Endocrinol Lett. 2002 Oct-Dec;23(5–6):459–82. 12500173

[35] Björkman L, Musial F, Alræk T, et al. Mercury, silver and selenium in serum before and after removal of amalgam restorations: results from a prospective cohort study in Norway. Acta Odontol Scand. 2023 Nov 16;81(4):298–310. 10.1080/00016357.2022.2143422 36383213

[36] Food and Drug Administration. Information for patients about dental amalgam fillings. US Food and Drug Administration; 2020 [updated 2020 Sep 24; cited 2023 Apr 25]. Available from: https://www.fda.gov/medical-devices/dental-amalgam-fillings/information-patients-about-dental-amalgam-fillings

[37] Kishi R, Doi R, Fukuchi Y, et al. Subjective symptoms and neurobehavioral performances of ex-mercury miners at an average of 18 years after the cessation of chronic exposure to mercury vapor. Mercury Workers Study Group. Environ Res. 1993 Aug;62(2):289–302. 10.1006/enrs.1993.1114 8344236

[38] Mathiesen T, Ellingsen DG, Kjuus H. Neuropsychological effects associated with exposure to mercury vapor among former chloralkali workers. Scand J Work Environ Health. 1999 Aug;25(4):342–50. 10.5271/sjweh.444 10505660

[39] Hill AB. The environment and disease: association or causation? Proc R Soc Med. 1965 May;58:295–300. 10.1177/003591576505800503 14283879 PMC1898525

[40] Rothman KJ, Greenland S. Hill’s criteria for causality. 2014. In: Wiley StatsRef: Statistics Reference Online [Internet]. [cited 2024 March 12] Available from: https://onlinelibrary.wiley.com/doi/abs/10.1002/9781118445112.stat05168.

[41] Sjursen TT, Lygre GB, Dalen K, et al. Changes in health complaints after removal of amalgam fillings. J Oral Rehabil. 2011 Nov;38(11):835–48. 10.1111/j.1365-2842.2011.02223.x 21517933 PMC3229679

[42] Stenman S, Grans L. Symptoms and differential diagnosis of patients fearing mercury toxicity from amalgam fillings. Scand J Work Environ Health. 1997;23 Suppl 3:59–63. 9456068

[43] Björkman L, Sjursen TT, Dalen K, et al. Long term changes in health complaints after removal of amalgam restorations. Acta Odontol Scand. 2017 Apr;75(3):208–19. 10.1080/00016357.2016.1278262 28093013

[44] Björkman L, Lundekvam BF, Laegreid T, et al. Mercury in human brain, blood, muscle and toenails in relation to exposure: an autopsy study. Environ Health. 2007;6:30. 10.1186/1476-069X-6-30 17931423 PMC2098763

