# Supplementary material for: Health complaints before and at one and five years after removal of dental amalgam restorations – data from a prospective cohort study in Norway
Source: Acta Odontol Scand. 2024 May 3;83:40260. doi: 10.2340/aos.v83.40260 (PMC11302653; doi:10.2340/aos.v83.40260)
Supplement: Supplementary file 1 — Health complaints before and at one and five years after removal of dental amalgam restorations – data from a prospective cohort study in Norway [file AOS-83-40260-s1.pdf]

Supplemental online material

Supplementary material has been published as submitted. It has not been copyedited or typeset by Acta Odontologica Scandinavica.

Supplemental Table S1. Items included in the Munich Amalgam Scale. Baseline mean, mean change score (Q1-Q2), standardized response mean (SRM) and 95% confidence interval are given for the Amalgam cohort. P-values for test of mean change=0 are given.

					95%CI	
**Item**	**Baseline mean**	**Mean change [a]**	**p-value [b]**	**SRM [c]**	**Upper**	**Lower**
Bleeding of the gums	0.78	0.13	0.255	0.21	-0.15	0.55
Fatigue	1.72	0.28	0.059	0.35	-0.01	0.70
Trembling	0.50	0.16	0.096	0.30	-0.05	0.66
Sleeplessness	1.25	0.22	0.198	0.23	-0.12	0.58
Getting tired fast	1.88	0.13	0.423	0.14	-0.21	0.49
Eye infection	0.25	0.03	0.786	0.05	-0.30	0.40
Susceptible to infection	0.66	0.16	0.407	0.15	-0.20	0.50
Loss of appetite	0.47	0.19	0.083	0.32	-0.04	0.67
Dyspnea	0.41	0.03	0.712	0.07	-0.28	0.41
Grinding one’s teeth	0.59	0.13	0.255	0.21	-0.15	0.55
Loosening of teeth	0.09	0.06	0.423	0.14	-0.21	0.49
Muscle weakness legs	1.00	0.22	0.129	0.28	-0.08	0.63
Reaction slowed down	0.47	-0.03	0.786	-0.05	-0.40	0.30
Lack of concentration	1.50	0.34	0.039	0.38	0.02	0.74
Memory disorders	1.22	0.06	0.645	0.08	-0.27	0.43
Intense nervousness	0.56	0.22	0.070	0.33	-0.03	0.69
Sadness	0.72	0.09	0.414	0.15	-0.20	0.49
Depressed mood	0.75	0.19	0.110	0.29	-0.07	0.64
Irritability	1.09	0.22	0.006	0.52	0.15	0.89
Worries - restlessness	1.06	0.47	0.005	0.53	0.16	0.90
Headache	1.06	0.00	1.000	0.00	-0.35	0.35
Burning sensations tongue	0.56	0.28	0.048	0.36	0.00	0.72
Dry mouth	0.91	0.16	0.344	0.17	-0.18	0.52
Metallic taste	0.91	0.88	<0.001	0.93	0.51	1.34
Dizziness	0.97	0.19	0.083	0.32	-0.04	0.67
Visual disorders	0.81	0.13	0.211	0.23	-0.13	0.58
Hearing disorders	0.72	0.06	0.423	0.14	-0.21	0.49
Cough attacks	0.41	0.00	1.000	0.00	-0.35	0.35
Arrythmia	0.75	0.28	0.027	0.41	0.05	0.77
Diarrhea	0.75	0.28	0.027	0.41	0.05	0.77
Vomiting	0.13	-0.03	0.662	-0.08	-0.42	0.27
Increased urge to toilet	1.09	0.28	0.027	0.41	0.05	0.77
Hair loss	0.41	0.13	0.458	0.13	-0.22	0.48
Skin rash	0.97	0.34	0.003	0.57	0.19	0.94
Skin itching	1.16	0.28	0.059	0.35	-0.01	0.70
Joint complaints	1.75	0.22	0.129	0.28	-0.08	0.63
Increased sweating	1.03	0.25	0.088	0.31	-0.05	0.66
Allergic reactions	1.06	0.28	0.071	0.33	-0.03	0.68
Sensitivity to cold, wind	1.25	0.41	0.005	0.54	0.16	0.91
Fluctuating mood	1.06	0.25	0.044	0.37	0.01	0.73
Indecision	0.97	0.28	0.027	0.41	0.05	0.77
Anxiety	0.78	0.25	0.044	0.37	0.01	0.73
Stress at the job	1.63	0.66	<0.001	0.83	0.43	1.23
Weight loss	0.44	0.19	0.136	0.27	-0.08	0.62
Overweight	0.50	0.09	0.263	0.20	-0.15	0.55
Constipation	0.59	0.06	0.601	0.09	-0.26	0.44
Abdominal pain	0.94	0.28	0.027	0.41	0.05	0.77
Tinnitus	1.19	0.19	0.110	0.29	-0.07	0.64
Menstrual disorders (n=19)	0.37	0.11	0.682	0.10	-0.43	0,64

Supplemental online material

Feeling of walking next to oneself	0.63	0.28	0.018	0.44	0.08	0.80

a) Baseline minus follow up value; A positive value indicates reduction of symptoms
b) 2-tailed significance from paired sample t-test
c) Standardized response mean (mean difference divided with the standard deviation of mean difference).

Supplemental online material

Supplemental Table S2. Mean intensity scores and standard errors (SE) for general health complaints at baseline and the follow-ups (Q2 and Q3) for the MUPS cohort (n=28). P-values calculated by linear mixed models for test of change from Q1 (H_0: Change score = 0).

	Q1		Q2			Q3		
	Mean	SE*	Mean	SE*	p	Mean	SE*	p
General symptoms								
Pain from muscles and joints	6.1	0.5	6.1	0.5	0.942	6.2	0.5	0.666
Gastrointestinal symptoms	3.2	0.5	2.9	0.5	0.551	2.7	0.5	0.360
Cardiovascular symptoms	1.4	0.3	1.2	0.3	0.414	1.3	0.3	0.658
General skin problems	1.7	0.4	1.4	0.4	0.514	1.7	0.4	0.896
Visual disturbances	2.2	0.4	2.7	0.4	0.311	2.6	0.4	0.507
Symptoms from ear/nose/throat	1.9	0.4	1.5	0.4	0.415	2.1	0.4	0.614
Fatigue	5.4	0.5	4.9	0.5	0.313	5.3	0.5	0.742
Dizziness	2.6	0.4	2.5	0.4	0.763	2.6	0.5	0.997
Headache	3.5	0.5	3.8	0.5	0.479	4.2	0.5	0.117
Memory problems	2.8	0.5	3.1	0.5	0.556	3.3	0.5	0.346
Difficult to concentrate	3.6	0.5	3.2	0.5	0.295	4.0	0.5	0.353
Anxiety/depression	2.1	0.4	2.0	0.4	0.828	2.4	0.4	0.611

* Delta method

Supplemental online material

Supplemental Table S3. Mean intensity scores and standard deviation (SD) for local and general health complaints at baseline (Q1) and the follow-ups (Q2 and Q3) for the Healthy cohort (n=12). Friedman's test was used for calculation of p-values to test if medians are equal (H_0: Q1=Q2=Q3).

	Q1		Q2		Q3		
	Mean	SD	Mean	SD	Mean	SD	p-value*
Local symptoms							
Intraoral burning sensation	0.0	0.0	0.2	0.6	0.4	1.4	0.778
Intraoral pain/tenderness	0.0	0.0	0.1	0.2	0.0	0.0	1.000
Taste disturbances	0.0	0.0	0.1	0.3	0.0	0.0	0.333
Intraoral stiffness/paresthesia	0.0	0.1	0.2	0.6	0.0	0.1	0.667
Dry mouth	0.3	0.6	0.1	0.2	0.5	0.9	0.099
Increased salivation/mucus	0.1	0.3	0.1	0.3	0.2	0.6	0.889
Orofacial burning sensation	0.1	0.3	0.1	0.2	0.0	0.0	0.667
Orofacial pain/tenderness	0.0	0.0	0.2	0.6	0.1	0.3	0.333
Orofacial stiffness/paresthesia	0.0	0.0	0.2	0.3	0.0	0.0	0.111
Orofacial skin problems	1.0	1.4	1.2	2.1	0.3	0.9	0.057
Pain from temporomandibular joints	0.3	0.9	0.4	0.7	0.3	0.8	0.898
General symptoms							
Pain from muscles and joints	1.6	2.0	1.8	1.8	2.6	2.1	0.292
Gastrointestinal symptoms	0.6	0.9	0.7	1.3	0.6	1.1	0.842
Cardiovascular symptoms	0.0	0.0	0.1	0.3	0.2	0.6	0.778
General skin problems	0.9	1.4	0.5	1.1	0.5	0.8	0.769
Visual disturbances	0.3	0.4	0.7	1.3	0.6	0.9	0.707
Symptoms from ear/nose/throat	0.0	0.0	0.0	0.1	0.1	0.3	0.111
Fatigue	0.7	1.0	0.4	0.7	0.3	0.6	0.879
Dizziness	0.0	0.0	0.1	0.2	0.2	0.4	0.444
Headache	0.2	0.4	0.4	0.5	0.2	0.6	0.194
Memory problems	0.0	0.0	0.3	0.7	0.1	0.3	0.333
Difficult to concentrate	0.1	0.3	0.3	0.5	0.3	0.4	0.457
Anxiety/depression	0.2	0.4	0.3	0.6	0.4	0.7	0.670

* Exact p-values
